# Engineering *Pseudomonas protegens* Pf‐5 to improve its antifungal activity and nitrogen fixation

**DOI:** 10.1111/1751-7915.13335

**Published:** 2018-11-20

**Authors:** Xiaoshu Jing, Qingwen Cui, Xiaochen Li, Jia Yin, Vinothkannan Ravichandran, Deng Pan, Jun Fu, Qiang Tu, Hailong Wang, Xiaoying Bian, Youming Zhang

**Affiliations:** ^1^ State Key Laboratory of Microbial Technology School of Life Science Shandong University‐Helmholtz Institute of Biotechnology Shandong University 266237 Qingdao China; ^2^ Jinan Yian Biology Institute Shandong Yian Biological Engineering Co. Ltd. Jinan 250100 China

## Abstract

In agricultural production, sustainability is currently one of the most significant concerns. The genetic modification of plant growth‐promoting rhizobacteria may provide a novel way to use natural bacteria as microbial inoculants. In this study, the root‐colonizing strain *Pseudomonas protegens* Pf‐5 was genetically modified to act as a biocontrol agent and biofertilizer with biological nitrogen fixation activity. Genetic inactivation of *retS* enhanced the production of 2,4‐diacetylphloroglucinol, which contributed for the enhanced antifungal activity. Then, the entire nitrogenase island with native promoter from *Pseudomonas stutzeri* DSM4166 was introduced into a *retS* mutant strain for expression. Root colonization patterns assessed via confocal laser scanning microscopy confirmed that GFP‐tagged bacterial were mainly located on root surfaces and at the junctions between epidermal root cells. Moreover, under pathogen and N‐limited double treatment conditions, the fresh weights of seedlings inoculated with the recombinant *retS* mutant‐*nif* strain were increased compared with those of the control. In conclusion, this study has innovatively developed an eco‐friendly alternative to the agrochemicals that will benefit global plant production significantly.

## Graphical abstract

In this study, the root‐colonizing strain *Pseudomonas protegens* Pf‐5 was genetically modified to act as a biocontrol agent and biofertilizer with biological nitrogen fixation activity. Under pathogen and N limited double treatment conditions, the fresh weights of seedlings inoculated with the recombinant were increased compared to those of the control. This study has innovatively developed an eco‐friendly alternative to the agrochemicals that will benefit global plant production significantly.

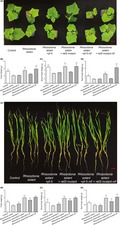

## Introduction

The extensive use of agrochemicals has led to harmful consequences for the environment and human health. Development of eco‐friendly sustainable biological agents is needed urgently to improve agricultural production globally. Plant growth‐promoting rhizobacteria (PGPRs) are a better alternative than agrochemicals for the promotion for plant growth and suppression of phytopathogens through both direct and indirect mechanisms (Khalid *et al*., 2013; Antweiler *et al*., 2017; Pandin *et al*., 2017). The direct mechanisms involve biological nitrogen fixation, phytohormone production, phosphate solubilization and iron chelating siderophore production (Vessey, 2003; Ryu *et al*., 2004; Ahemad, 2014), while the major indirect mechanisms include biocontrol activity through the secretion of antifungal metabolites (Kim *et al*., 2011; Bhattacharyya and Jha, 2012), and the induction of plant defence systems such as systemic acquired resistance (SAR) and induced systemic resistance (ISR; Lugtenberg and Kamilova, 2009). Some PGPR have biocontrol properties, while other strains effectively uptake nutrients to positively regulate plant growth and development. Finding strains that effectively fulfil both roles is uncommon. Thus, combining biocontrol properties and nutrient production activity into one PGPR via genetic manipulation may be an innovative approach comparable to the use of natural bacteria as inoculants.

*Pseudomonas fluorescens* is well‐characterized PGPR known for its production of a broad spectrum of secondary metabolites and root colonization of wide range of hosts (Table S1; Budzikiewicz, 1993; Lopes *et al*., 2017, 2018; Khalid *et al*., 2004, 2013; Weber *et al*., 2015). *Pseudomonas protegens* Pf‐5 (formerly *Pseudomonas fluorescens* Pf‐5) was isolated from the cotton rhizosphere, which typically protects plants from diseases caused by plant pathogens (Howell and Stipanovic, 1979, 1980; Xu and Gross, 1986; Rodriguez and Pfender, 1997; Sexton *et al*., 2017). At least 6% of the genome of *P. protegens* Pf‐5 is involved in secondary metabolite production (Paulsen *et al*., 2005), including that of pyrrolnitrin (Howell and Stipanovic, 1979, 1980), 2,4‐diacetylphloroglucinol (Nowak‐Thompson *et al*., 1994), pyoluteorin (Howell and Stipanovic, 1980), hydrogen cyanide (Kraus and Loper, 1992), rhizoxin analogues (Loper *et al*., 2008) and toxoflavin (Philmus *et al*., 2015). The production of these antibiotics requires the Gac/Rsm signal transduction pathway (Haas and Défago, 2005; Lapouge *et al*., 2008). The GacS/A system, a two‐component regulatory system, comprises a sensor kinase GacS, a transcriptional regulator GacA and small regulatory RNA (Brencic *et al*., 2009). Upon autophosphorylation, GacS senses a signal and transfers its phosphate to GacA (Brencic *et al*., 2009). Phosphorylated GacA then positively regulates the expression of small regulatory RNAs that bind to the repressor proteins RsmA/E, thereby relieving translational repression on target genes encoding biocontrol factors (Valverde *et al*., 2003; Kay *et al*., 2005; Reimmann *et al*., 2005; Lapouge *et al*., 2008). RetS, a sensor kinase, has been reported to negatively regulate the production of antifungal secondary metabolites via the Gac/Rsm pathway in *P. aeruginosa*,* P. protegens* CHA0 and *P. fluorescens* FD6 (Goodman *et al*., 2004; Laskowski and Kazmierczak, 2006; Humair *et al*., 2009; Zhang *et al*., 2015). RetS sensors have a negative effect on the expression of GacA‐controlled sRNA genes. The combination of RetS and GacS has been proven to prevent the phosphorylation of GacA, which subsequently leads to obstructions of the Gac/Rsm pathway (Goodman *et al*., 2004; Gooderham and Hancock, 2009; Workentine *et al*., 2009; Quecine *et al*., 2015). As a result, antibiotic biosynthetic genes such as 2,4‐DAPG are downregulated and the production of related antibiotics is reduced (Kay *et al*., 2005; Reimmann *et al*., 2005). Accordingly, a *retS* mutant was found to have strongly enhanced production of biocontrol factors.

Biological nitrogen fixation, the process in which N_2_ is converted to NH_3_ by bacteria, offers a natural way of providing nitrogen to plants. The inoculation of non‐legume crop plants with diazotrophic bacteria is an attractive option for reducing chemical nitrogen fertilizer requirements. Since the *nif* cluster was initially identified in *Pseudomonas stutzeri* A1501 was found to be essential for nitrogen fixation, several nitrogen‐fixing strains belonging to *P. stutzeri* have recently been isolated and characterized (Desnoues *et al*., 2003; Yan *et al*., 2008; Venieraki *et al*., 2011; Yu *et al*., 2011; Pena *et al*., 2013). Bioinformatics analysis has demonstrated that all putative *nif genes* in diazotrophic *Pseudomonas* species are anchored in an intergenic region with flanking regions designated by distinct repeats patterns (Venieraki *et al*., 2014), indicating that the *nif genes* can be acquired by horizontal transfer among microorganisms. *P. stutzeri* DSM 4166, which is highly similar overall to *P. stutzeri* A1501 (Desnoues *et al*., 2003; Yan *et al*., 2008), was isolated from the rhizosphere of a *Sorghum nutans* cultivar in Germany (Yu *et al*., 2011). This strain contains 58 genes clustered in a 49‐kb putative nitrogen fixation island and harbours all of the genes required for complete denitrification and nitrate assimilation (Yu *et al*., 2011); however, it has limited production of secondary metabolites (Table S1; Weber *et al*., 2015).

*P*. *protegens* Pf‐5 has only biocontrol properties, while *P. stutzeri* DSM 4166 is an effective nitrogen fixer. The transfer of nitrogen fixation activity into root‐colonizing *P*. *protegens* Pf‐5, with its biocontrol properties, would improve its agricultural performance. Horizontal transfer of the *nif* genes among microorganisms confers the ability to fix nitrogen in a non‐nitrogen‐fixing heterologous host (Dixon and Postgate, 1971; Setten *et al*., 2013; Fox *et al*., 2016). Our group developed a Red/ET recombineering‐mediated direct cloning technology that has been successfully used to efficiently clone and heterologously express large biosynthetic gene clusters in multiple hosts (Fu *et al*., 2012; Bian *et al*., 2015; Wang *et al*., 2016, 2017). The entire nitrogenase island from the digested genomic DNA of *P. stutzeri* DSM4166 was cloned into a Bacterial Artificial Chromosome (BAC) vector, followed by the addition of a cassette‐containing conjugation and MycoMar transposition elements (Yu *et al*., 2018), which can facilitate the cloning and transfer of the *nif* island among *Pseudomonas* species (Fu *et al*., 2008). Thus, the entire nitrogenase island from *P. stutzeri* DSM4166 was transferred to *P*. *protegens* Pf‐5 to construct a recombinant strain with nitrogen fixation and biocontrol properties.

In this study, we generated an engineered strain of *P. protegens* Pf‐5 with nitrogen fixation and enhanced antifungal activities. We knocked out the *retS* gene and elucidated its role in the negative control of antibiotics biosynthesis in strain Pf‐5. Antibiotic production was improved in the *retS* mutant, as illustrated by a distinct zone of inhibition when grown with *Rhizoctonia solani*. A recombinant nitrogen‐fixing *Pseudomonas* strain was obtained by integrating of the *nif* genes from *P. stutzeri* into the mutant genomes randomly. The recombinant showed high levels of nitrogenase activity. Further, the colonization patterns of the recombinant strains in roots were observed using confocal laser scanning microscope (CLSM). GFP‐labelled bacterial colonized root hairs and root surfaces as well as the junctions between epidermal root cells. Interestingly, the recombinant also increased seedlings fresh weights under pathogen and N‐limited double treatment conditions. This study provides a new approach for agricultural sustainability.

## Results and discussion

### Indirect regulation of antibiotic biosynthesis and antifungal activity by the *retS* gene

**Figure 1:**
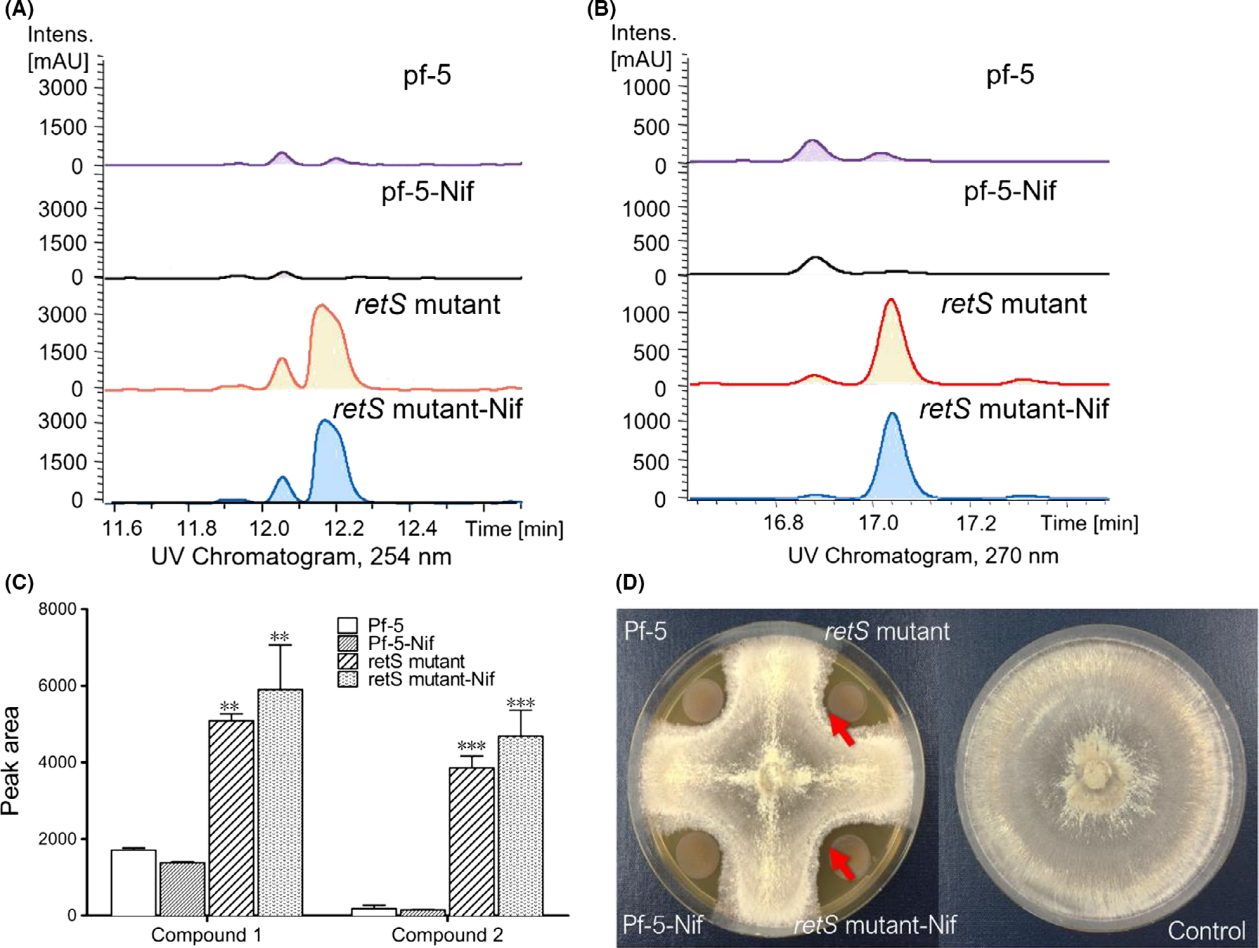
Influence of the *retS* gene on the production of antibiotics in *P. protegens* Pf‐5. HPLC spectrum of compounds 1 (A) and 2 (B) from the Pf‐5, *retS* mutant, Pf‐5‐*nif* and *retS* mutant‐*nif* strains (C) Relative production of compounds 1 and 2. (D) Zones of inhibition (the arrowheads) on *Rhizoctonia solani*. Each column is the mean of three independent measurements at least. **P < 0.01 and ***P < 0.001 according to ANOVA with Dunnett as post doc test.

**Figure 2:**
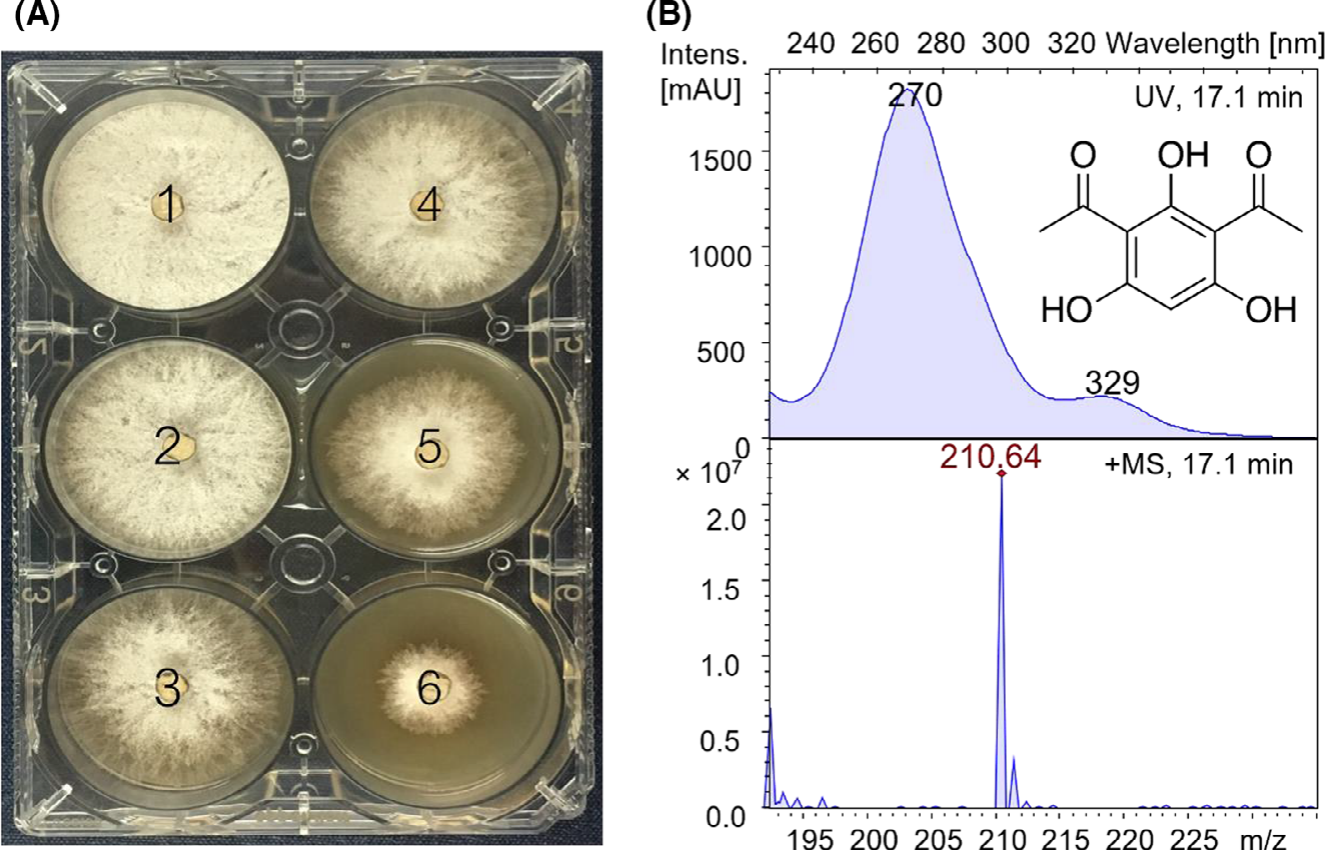
Inhibition of *Rhizoctonia solani* and HPLC spectrum of compound 2.A. Susceptibility of *Rhizoctonia solani* to C2 in 6‐well plates. The number 1‐6 indicates the C2 concentrations of: 0, 5, 10, 15, 50 and 100 μg ml^−1^ respectively.B. HPLC spectrum, MS spectrum and UV visible spectrum of compound 2.

In *P. protegens*, RetS sensors act as an antagonist of the GacS/GacA system (Goodman *et al*., 2004; Laskowski and Kazmierczak, 2006; Humair *et al*., 2009; Zhang *et al*., 2015). To investigate the impact of *retS* on antibiotic production and antifungal activity in strain Pf‐5, a *retS* mutant was created via a recombineering method based on native phage protein pairs in *Pseudomonas* (unpublished data; Zhang *et al*., 1998; Yin *et al*., 2015). The production of antibiotics by *P. protegens* Pf‐5 and *retS* mutants was evaluated by HPLC/MS (Fig. S1). Compared with the wild‐type strain, two large peaks appeared at 12.2 min and 17.1 min in the *retS* mutant extracts (Fig. 1A and B; Fig. S1). The compounds C1 and C2 were vastly increased by threefold~fourfold (C1), 20~30‐fold (C2) in the *retS* mutant compared with the wild‐type strain (Fig. 1C). The antifungal activity of the *retS* mutant was more significant, as revealed by the zone of inhibition of *Rhizoctonia solani* (Fig. 1C and D), compared with those of the wild‐type strain (Fig. 1D). Antifungal activity was also assessed with *Botrytis cinereal*; however, the activity against *Botrytis cinereal* was unaffected in the *retS* mutant (data not shown). To investigate the antifungal activity of the produced compounds against *Rhizoctonia solani*, compounds **C1** and **C2** were purified by preparative HPLC (Fig. S2) and susceptibility testing was performed (Fig. 2). Compound **C2** showed excellent growth inhibition of *Rhizoctonia solani* (Fig. 2A), while compound **C1** did not have any significant antifungal activity against the tested fungi (date not shown). Compound **C2** had a large peak at 270 nm and a small peak at 330 nm, identical to those of 2,4‐DAPG (Fig. 2B); in addition, the *m*/*z* value and maximum absorption wavelength data for **C2** were also comparable to 2,4‐DAPG. Enhanced the production of 2,4‐DAPG and antifungal activity was further revealed by the inhibition of *Rhizoctonia solani* growth in 6‐well plates (Figs 1 and 2). The production of pyoluteorin (19.5 min; Fig. S3), orfamide A (26.7 min; Fig. S4), orfamide B (27.3 min; Fig. S4) and orfamide C (26.1 min; Fig. S4) were also identified by HPLC/MS. However, no significant differences in the production of these compounds were found in the *retS* mutant. These results suggest that *retS* could increase 2,4‐DAPG production in *P. protegens* Pf‐5, similar to *P. protegens* CHA0 and *P. fluorescens* FD6 (Humair *et al*., 2009; Zhang *et al*., 2015). To obtain multifunctional *P. protegens* capable of nitrogen fixation and enhanced antifungal production, the *retS* mutant Pf‐5 was used as the chassis for the introduction of *nif* genes.

### Genomic analysis of *nif* genes and nitrogenase activity in *P. protegens* Pf‐5

**Figure 3:**
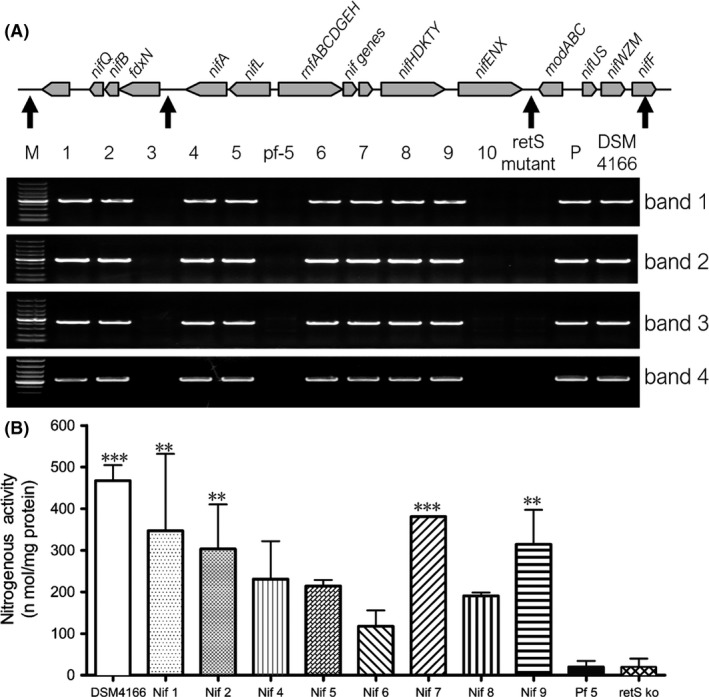
Genomic PCR detection of the *nif* genes (A) and the relative nitrogenase activity (B) in *P. protegens*.A. The physical region (arrowheads) and the PCR products (bands 1–4) of the four pairs check primers. The *nif* genes in the wild‐type *P. protegens* (lane 1–5) and retS mutant (lane 6–10). Lane M: DL5000 ladder. Lane P: BAC containing the *nif* genes.B. Relative nitrogenase activity. DSM 4166 was used as a positive control. Pf‐5 and the *retS* mutant were used as negative control. Each column is the mean of three independent measurements at least. Bars represent standard error of the mean. **P < 0.01 and ***P < 0.001 according to ANOVA with Dunnett as post doc test.

Bioinformatics analysis demonstrated that the *nif* genes could be acquired by horizontal transfer among microorganisms (Venieraki *et al*., 2014). Direct cloning via Red/ET recombineering is an updated method for horizontal gene transfer (Zhang *et al*., 2000; Wang *et al*., 2016, 2017), in which non‐nitrogen‐fixing wild‐type *P. protegens* could be converted to nitrogen fixers through plausible lateral gene transfer (Setten *et al*., 2013; Fox *et al*., 2016). The *nif* genes were introduced into the Pf‐5 and *retS* mutant strains via conjugation and MycoMar transposition (Fig. S5). Recombinant containing *nif* genes was named Pf‐5‐*nif* or *retS* mutant‐*nif* respectively. Genomic PCR analysis confirmed the presence of the *nif* genes in these *P. protegens* strains (Fig. 3A). In addition, the *retS* mutant, Pf‐5‐*nif* and *retS* mutant‐*nif* displayed similar growth characteristics as the wild‐type strain on LB medium (Fig. S6), indicating that the growth rate was not affected by the *retS* or *nif* genes.

To understand whether the *nif* genes functioned properly in the heterologous host, we analysed the nitrogenase activity of the recombinants by measuring acetylene reduction in L‐medium (Setten *et al*., 2013) without nitrogen under microaerobiosis. The engineered strains Pf‐5 *nif* or *retS* mutant‐*nif* showed significant nitrogenase activity (Fig. 3B), whereas no nitrogenase activity was observed in the wild‐type Pf‐5 or the *retS* mutant strains. In addition, high ammonium production in the L‐medium of the Pf‐5‐*nif* strain was associated with the activity (Fig. S7). The recombinants displayed different levels of nitrogenase activity due to integration site for the *nif* genes. Compared with other recombinants, recombinant strains Pf‐5‐*nif*‐2 and *retS* mutant‐ *nif*‐7 showed remarkable nitrogenase activity, indicating that the *nif* genes were more efficiently expressed in these strains. To assess the stability of the integrated *nif* genes, recombinant strains Pf‐5‐*nif*‐2 and *retS* mutant‐ *nif*‐7 were sequentially subcultured in LB medium 30 times, the *nif* genes were stably present, as confirmed by genomic PCR analysis (during this process, the plasmid expressing the Red/ET recombineering system based on native phage protein pairs was removed, and its absence was verified by double streak). Next, the localization patterns and phenotypes of plants inoculated with the recombinant strains were examined.

### Root localization patterns and phenotype analysis of the engineered *P. protegens* Pf‐5 strains on *Arabidopsis* of seedlings

**Figure 4:**
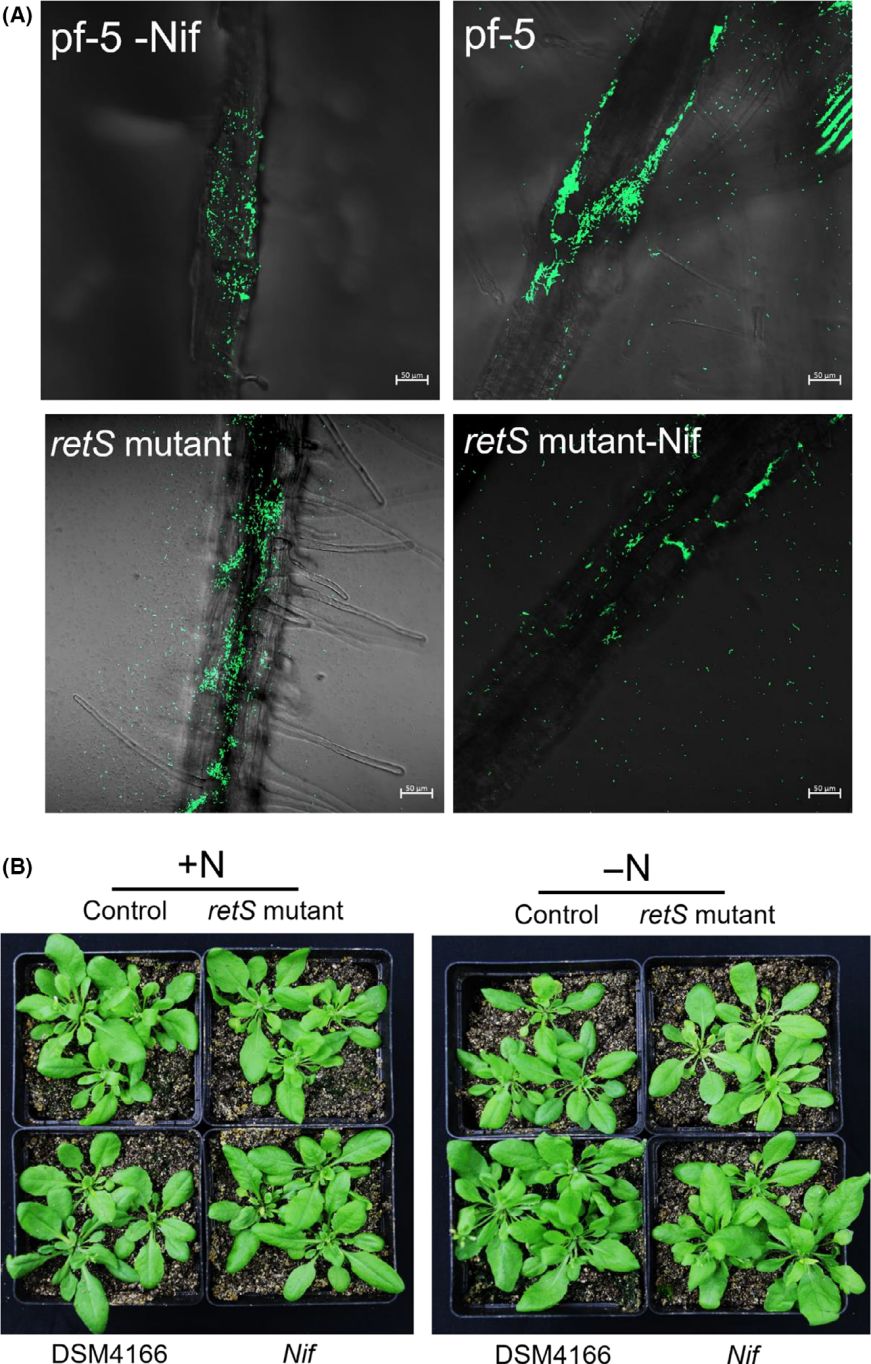
The colonization of GFP‐labelled *P. protegens* on *Arabidopsis* roots (A) and Growth phenotype of *Arabidopsis* with or without nitrogen (B). Surface‐sterilized seeds of *Arabidopsis* were sown on 1/2 MS semisolidified medium. The colonization patterns of elongation zone after 7 days of seeds inoculation. At 7 days post germination, the *Arabidopsis* was grown in cultures containing a mixture of peat and vermiculite (3:1 v/v) and were treated with *P. protegens* with or without nitrogen source. Bars = 50 μm.

Efficient root colonization by plant rhizobacteria is essential for biocontrol activity. The effects of the *retS* and *nif* genes on roots colonization of the model plant – *Arabidopsis* were assessed first. A vector with the green fluorescent protein gene (GFP) was constructed and introduced into the Pf‐5, *retS* mutant, Pf‐5‐*nif* and *retS* mutant‐*nif* strains (Fig. S8). The root colonization patterns were then observed using CLSM after inoculating *Arabidopsis* seeds with the GFP‐tagged bacteria (Fig. 4). GFP was efficiently expressed in the *Pseudomonas* strains (Fig. S8), and no fluorescence signal was detected in *Arabidopsis* roots controls inoculated with the unmodified strain (Fig. S8). GFP‐tagged bacteria were visualized on *Arabidopsis* roots, indicating that the bacterial successfully survived and colonized the roots (Fig. 4A).

The colonization pattern of the recombinant was expected to be correlated with the productivity. Thus, the engineered *retS* mutant‐*nif* was used to analyse the impact of the *nif* genes on plant growth. *Arabidopsis* was grown in cultures treated with nitrogen (+N) or without nitrogen (−N) and was inoculated with DSM 4166 (positive), the *retS* mutant (negative) or the *retS* mutant‐*nif* (engineered bacteria), individually. In the absence of nitrogen source, the rosette diameter of plants inoculated with the recombinant strain increased more than that of plants inoculated with the *retS* mutant strain for both the mixture cultures of peat and vermiculite (3:1 v/v; Fig. 4B) and 100% vermiculite cultures (Fig. S9); however, a more significant increase occurred in the culture containing 100% vermiculite (Fig. S9). Nevertheless, no differences were observed between *Arabidopsis* plants inoculated with the control and the *retS* mutant‐*nif* strains when nitrogen was present (Fig. 4B). Considering the nutrient contents of the cultures, these results indicated that this recombinant strain can retain at least some of negative regulation of the nitrogenase activity under excess nitrogen conditions. Furthermore, the *nif* genes in the recombinant strain promoted plant growth, as shown in the previous study (Setten *et al*., 2013; Fox *et al*., 2016).

### Roots localization patterns and phenotype analysis of the engineered *P. protegens* Pf‐5 strains on wheat and cucumber

**Figure 5:**
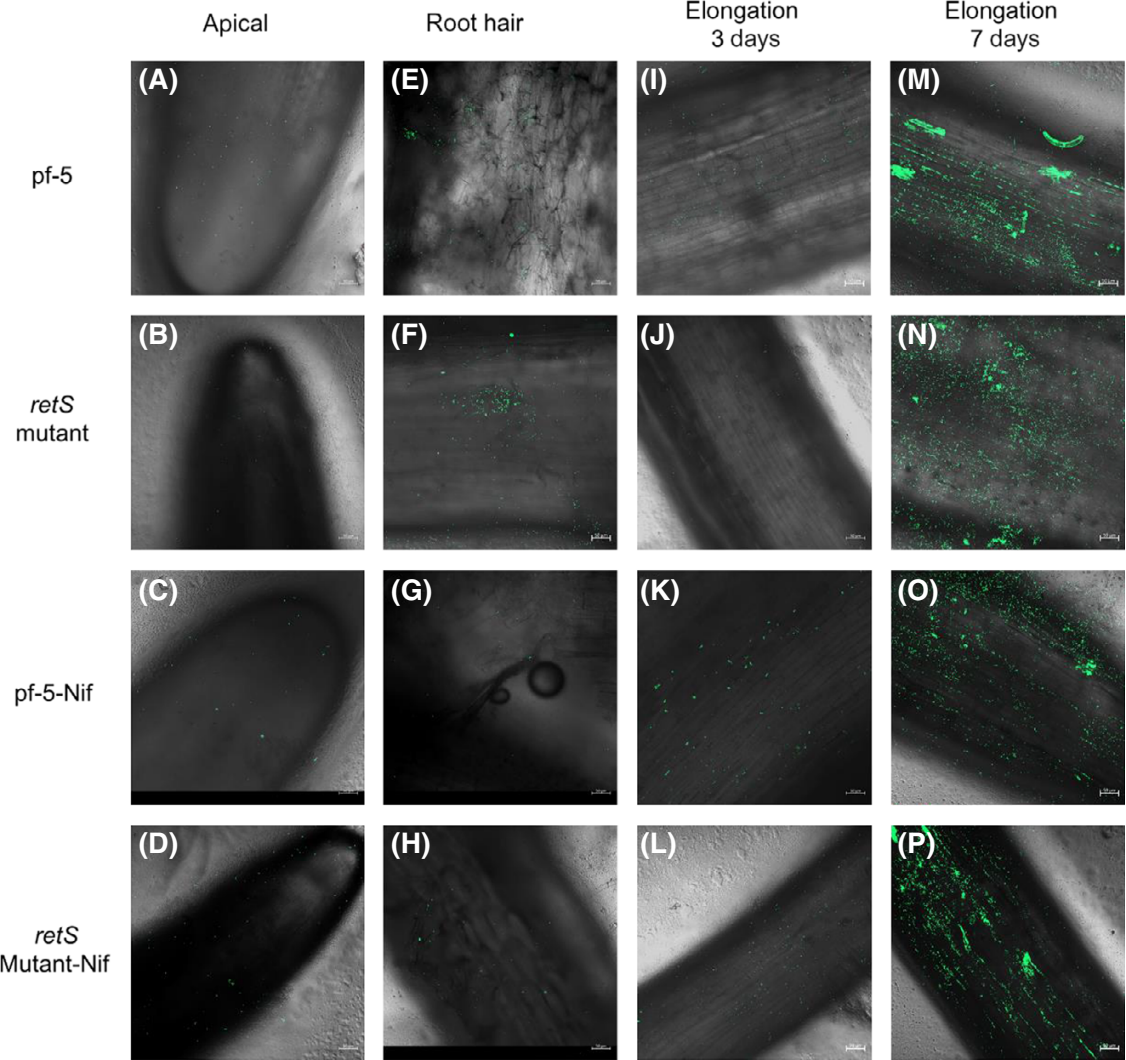
The colonization of GFP‐labelled *P. protegens* on cucumber roots.A–D. The colonization patterns of apical 3–5 days after inoculation.E–H. The colonization patterns of root hair 3–5 days after inoculation.I–L. The colonization patterns of elongation zone 3–5 days after inoculation.M–P. The colonization patterns of elongation zone 7 days after inoculation. Bars = 50 μm.

**Figure 6:**
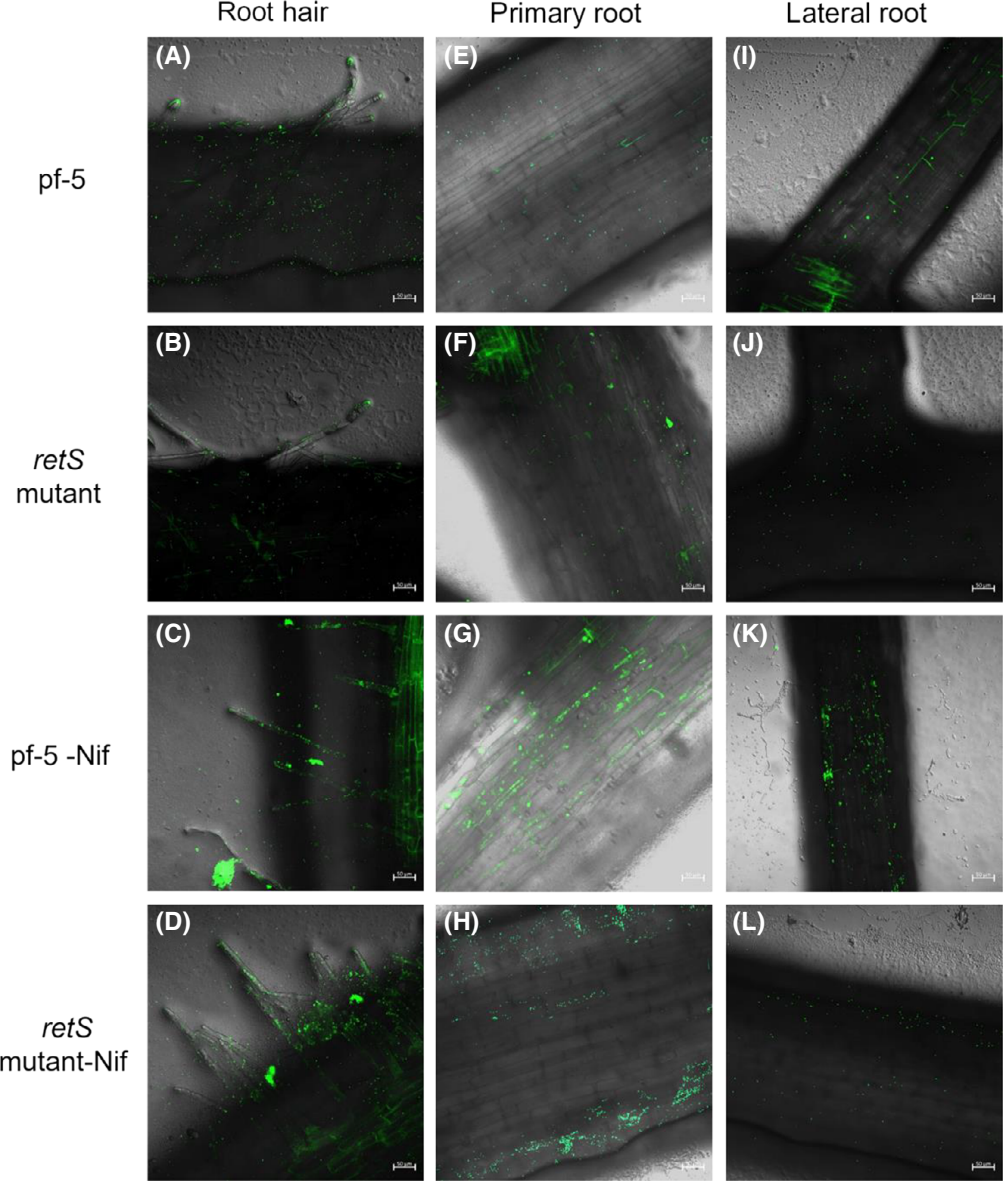
The colonization of the GFP‐labelled *P. protegens* on wheat roots.A–D. The colonization patterns of root hair after 7 days after inoculation.E–H. The colonization patterns of primary root 7 days after inoculation.I–L. The colonization patterns of lateral root 7 days after inoculation. Bars = 50 μm.

To further extend potential nitrogen‐fixing ability to major cereal crops and vegetable, the effects of the *retS* and *nif* genes on root colonization were also studied on wheat (monocotyledons, crop) and cucumber (dicotyledon, vegetable) under growth room conditions. In the wheat and cucumber roots controls, no fluorescence signal was detected (Fig. S8). In cucumber, GFP‐tagged bacteria were visualized on root surfaces after 3–5 days after inoculation (Fig. 5A–P). The bacterial were found to colonize on root tips and root hairs via single‐cell and small‐aggregate modes of colonization (Fig. 5A–H). Seven days after inoculation, more bacteria were localized on the root surface, and dispersed large‐aggregate colonization was observed (Fig. 5M–P), indicating that the bacterial were successfully survived and colonized the roots. In addition, a linear distribution of bacterial was also observed at the junctions between epidermal root cells (Fig. 5M–P). Consistent with the results obtained in cucumber, GFP‐tagged bacterial also localized on the root hair of wheat (Fig. 6A–D). Furthermore, bacterial colonized the primary roots (Fig. 6E–H) and lateral roots (Fig. 6I–L). These results showed that the *retS* and *nif* genes had no influence on colonization pattern when adapted to different hosts (Figs 4, 5, 6). In addition, the colonization patterns of the *retS* mutant‐GFP, *nif*‐GFP and *retS* mutant‐*nif*‐GFP strains were analogous to that of the wild‐type Pf‐5 (Figs 4, 5, 6).

Phenotype analysis of wheat and cucumber to assess the nitrogen‐fixing ability of the strains was first performed under N‐limited conditions. Improvement in the seedlings was reflected in their fresh weights. Without nitrogen, plants inoculated with the recombinant strain expressing *nif* genes showed significantly higher productivity than the *retS* mutant strains and the wild‐type pf‐5 (Fig. S10). The fresh weights were increased by 74%, 45% and 135% in the cucumber seedlings, shoots and roots respectively (Fig. S10B–D). The fresh weights of wheat seedlings, shoots and roots were similarly increased by 57%, 53% and 131% respectively (Fig. S10F–H).

The combination of nitrogen‐fixing ability and antifungal activity was further studied on the major cereal crop (wheat) and vegetable (cucumber) under growth room conditions. Prior to the inoculation of *P. protegens*, wheat/cucumber seedlings were grown in pots infected by *Rhizoctonia solani* and watered by 1/4 Hoagland's nutrient solution without nitrogen. The wheat/cucumber plants were then inoculated with the Pf‐5, *retS* mutant, Pf‐5‐*nif* or *retS* mutant‐*nif* strain.

**Figure 7:**
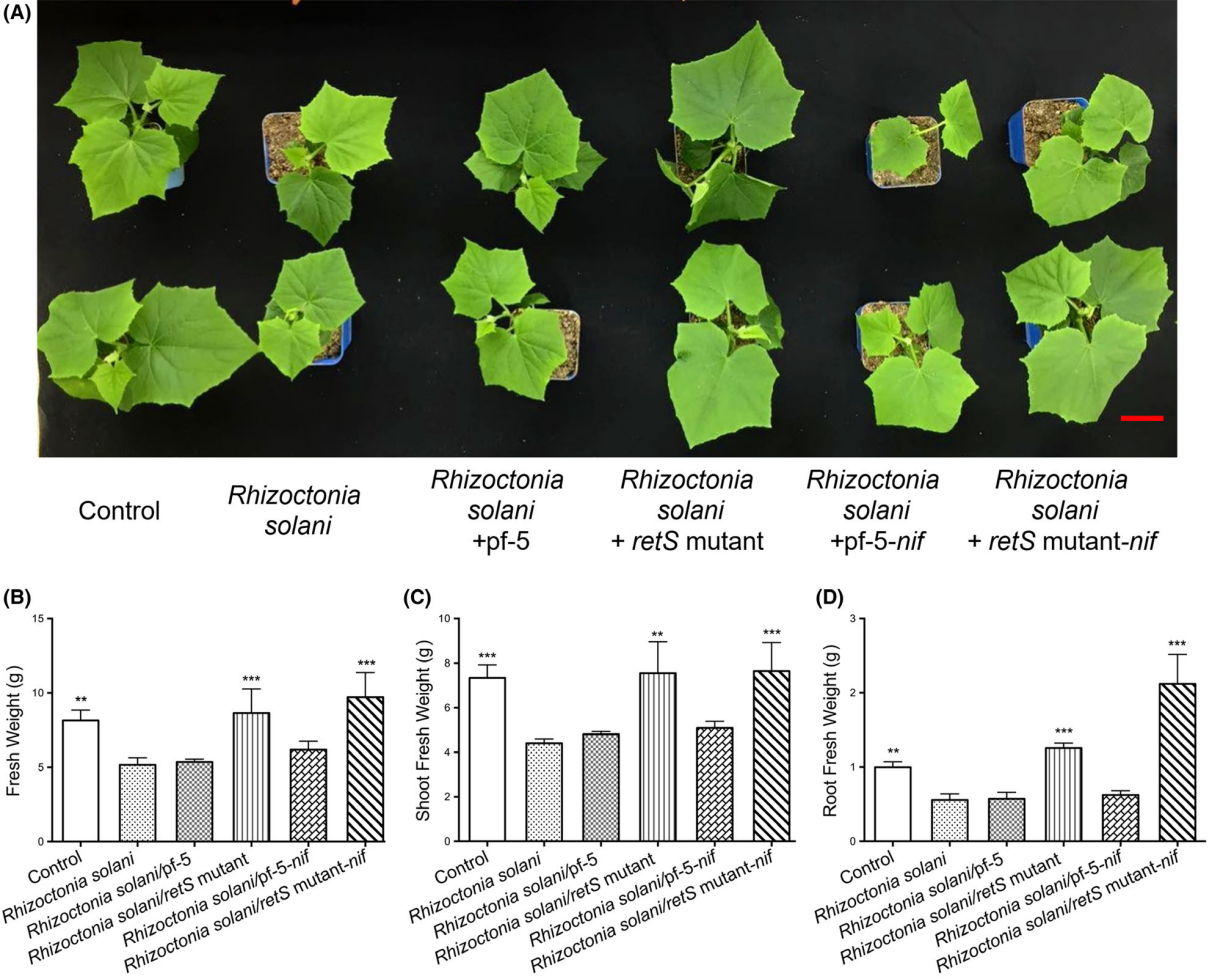
Growth phenotype of cucumber under pathogen and N‐limited double treatment condition. Surface‐sterilized cucumber seeds were sown on 1/2 MS semisolidified medium. At 7 days post germination, seedlings were grown in cultures infected with *Rhizoctonia solani* in 1/4 Hoagland's nutrient solution without nitrogen. After 3 days of cocultivation, the seedlings were inoculated with *P. protegens*. Photographs were taken 14 days after inoculation, and fresh weights were measured. A, B, C and D show plant performance (A), fresh weights of seedlings (B), shoots (C) or roots (D). Each column is the mean of six independent measurements. Bars represent standard error of the mean. Bars = 2 cm. **P < 0.01 and ***P < 0.001 according to ANOVA with Dunnett as post doc test.

In cucumber, *Rhizoctonia solani* inoculation caused the fresh weights of the seedlings, shoots and roots to decrease by 37%, 40% and 44%, respectively, compared with plants not incubated with *Rhizoctonia solani* inoculation (Fig. 7). Similar to the *in vitro* results (Fig. 2), the *retS* mutant‐treated plants demonstrated that biocontrol potential against *Rhizoctonia solani* (Fig. 7). The *retS* mutant with or without *nif* genes exhibited protective effects in the presence of *Rhizoctonia solani*, resulting in increased fresh plant, shoot and root weights. Conversely, the effects of the Pf‐5 or the Pf‐5‐*nif* strains were sporadic (Fig. 7). Compared with the healthy control, seedling treatment with *retS* mutant had slightly enhanced the fresh biomass, especially in the root (Fig. 7D). In addition, the *retS* mutant‐*nif* strain resulted in a lower shoot/root ratio than that of the control, and it increased the fresh plant, shoot and root weights by 19%, 4% and 113% respectively (Fig. 7B–D).

**Figure 8:**
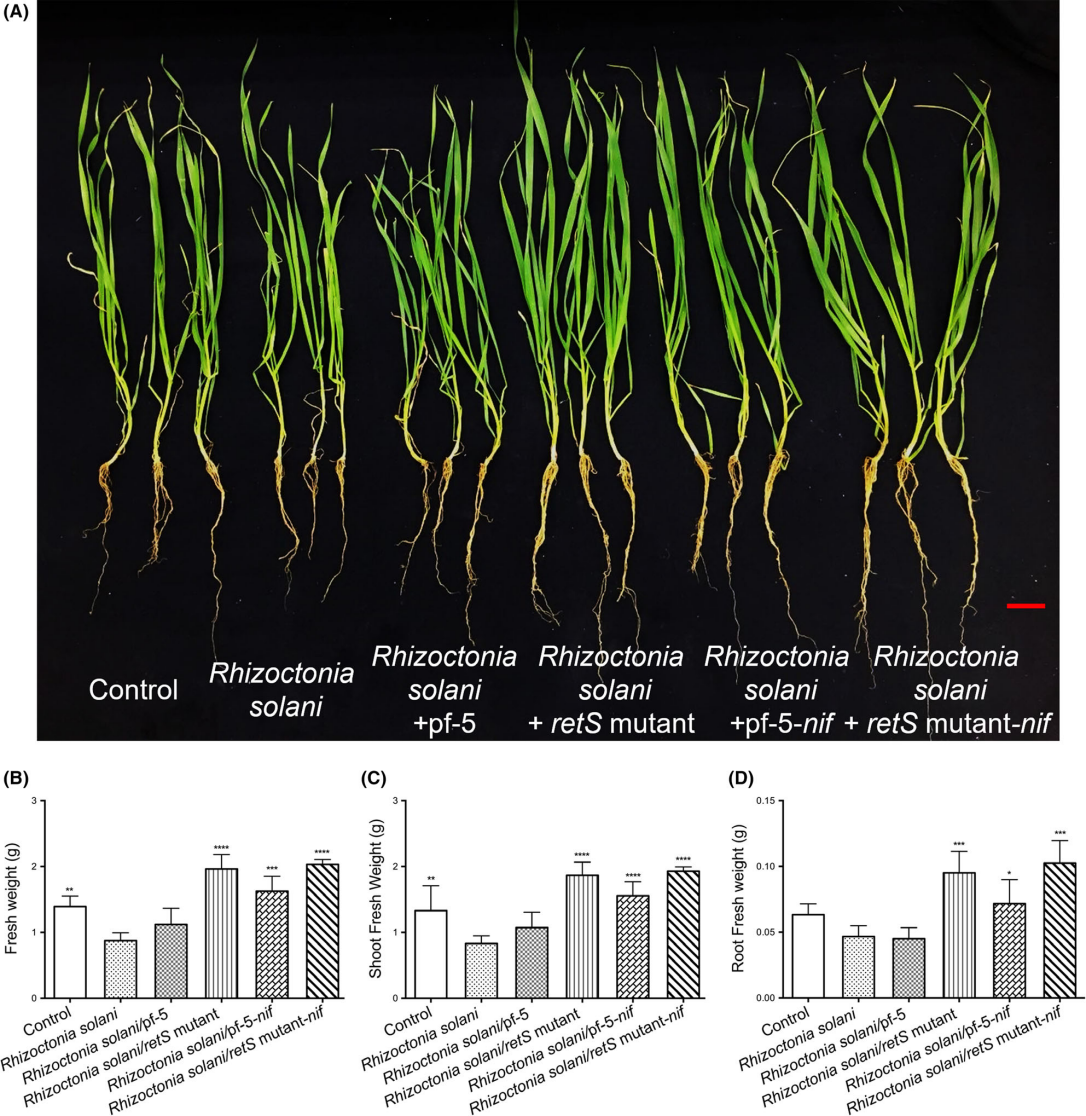
Growth phenotype of wheat under pathogen and N‐limited double treatment condition. Surface‐sterilized wheat seeds were sown on 1/2 MS semisolidified medium. At 7 days of postgermination, seedlings were grown in cultures infected with *Rhizoctonia solani* under 1/4 Hoagland's nutrient solution without nitrogen. After 3 days of cocultivation, the seedlings were inoculated with *P. protegens*. Photographs and fresh weights were taken after 14 days of cocultivation with the inoculated treatment. A, B, C and D show plant performance (A), fresh weights of seedlings (B), shoots (C) or roots (D). Each column is the mean of 6 independent measurements. Bars represent standard error of the mean. Bars = 1 cm. *P < 0.05, **P < 0.01, ***P < 0.001 and ****P < 0.0001 according to ANOVA with Dunnett as post doc test.

For wheat, inoculation with *Rhizoctonia solani* significantly reduced the fresh plant, shoot and root by 37%, 38% and 22%, respectively, which also were the lowest fresh weights observed (Fig. 8). Similar to cucumber, the fresh weights of pathogen‐inoculated wheat were unaffected by inoculation with wild‐type Pf‐5 (Figs 7 and 8). However, the fresh weights increased significantly among plants inoculated with the *retS* mutant, Pf‐5‐*nif* and *retS* mutant‐*nif* strains in the presence of pathogen (Fig. 8B–D). For the *Rhizoctonia solani* treatments, inoculation with the Pf‐5‐*nif* increased the fresh seedling, shoot and root weights by 86%, 87% and 53% respectively (Fig. 8B–D). Moreover, the *retS* mutant‐*nif* strain had the greatest overall effects in the presence of *Rhizoctonia solani* in relation to the control (Fig. 8B–D).

Inoculation with *Rhizoctonia solani* significantly affected the growth of wheat and cucumber seedlings, compared with the pathogen‐inoculated and uninoculated control under N‐limited condition (Figs 7 and 8). Consistent with the antifungal activity against *Rhizoctonia solani in vitro*, the effects of nitrogen limitation were significantly suppressed by the engineered *P. protegens* Pf‐5 strain we constructed in this study. Compared the control, the fresh weights were unaffected with inoculation of the engineered *P. protegens* Pf‐5 strain under the pathogen treatments (Figs 7 and 8). Furthermore, these seedlings were healthy and vigorous under pathogen and N‐limited double treatment condition (Figs 7 and 8). These results indicate that the engineered *P. protegens retS* mutant‐*nif* strain may act as a biofactory for the biocontrol of plant pathogens and biological fixation of nitrogen.

## Conclusions

Numerous studies on the use of PGPRs for plant growth promotion have been reported, but interest in the potentials of microbial inoculants for agriculture has increased within the last few years. Precise and fluent genetic manipulation have greatly expanded recombinant DNA technology for genome engineering (Zhang *et al*., 1998; Yin *et al*., 2015). Direct cloning via a Red/ET recombineering method based on phage‐encoded homologous recombination systems is an updated method for horizontal gene transfer (Zhang *et al*., 1998, 2000; Wang *et al*., 2016, 2017) that can be applied to other host strains or to extend antimicrobial spectrum, which could open new avenues for the development of sustainable agriculture. Despite the great potential of these genetically modified strains, understanding its complexities is essential. For biological nitrogen fixation, for example, the activity of the engineered strain could potentially be affected by the integration site of the genes as well as by the soil types and soil oxygen levels under agricultural conditions. In this regard, refactoring the *nif* genes to unlock potential constraints would be interesting and could expand the use of nitrogen‐fixing inoculants to diverse plants and soils.

## Experimental procedures

### Microorganisms, plasmids, primers and growth conditions

*Escherichia coli* GB05‐dir and GB05‐red were used for recombineering were grown in LB medium supplemented with appropriate antibiotics (chloramphenicol 15 μg ml^−1^, ampicillin 100 μg ml^−1^ and gentamicin 4 μg ml^−1^). *E. coli* ET12567/pUZ8002 was grown in LB broth at 37°C overnight with 50 μg ml^−1^ kanamycin. *P. protegens* Pf‐5 was grown in LB broth at 28°C overnight with shaking. After conjugation, PMM (K_2_HPO_4_, 8 g l^−1^; KH_2_PO_4_, 5 g l^−1^; (NH_4_)_2_SO_4_, 1 g l^−1^; succinic acid, 6.6 g l^−1^; pH 7.0. After autoclaving, 20 mM MgSO_4_ was added) medium with appropriate antibiotics was used to select for recombinants. All the strains, plasmid and primers used in this study are listed in Table S2.

### Construction of the *retS* deletion mutant

A gene removal system containing a gentamicin resistance gene and a Cre‐lox66/lox71 cassette was used to replace the *retS* gene in the genome of *P. protegens* Pf‐5 using Red/ET recombineering. PCR products containing a 100‐bp sequence homologous to the *retS* gene at their 5′ ends and a 22‐bp PCR primers at their 3′ ends were amplified and electroporated by eporator (1250 V, 5 ms, Eppendorf Corporation) into a *P. protegens* Pf‐5 expressing Red/ET recombineering system based on the native phage protein pairs (novel recombineering systems for *Pseudomonas*, our unpublished data; Zhang *et al*., 2000; Yin *et al*., 2015). After deleting the lox66/lox71‐flanked genta^R^ gene, the *retS* mutation was verified by PCR using primers *retS* check 1 and *retS* check 2 that were designed upstream and downstream of the *retS* gene.

### Construction of the GFP vector

The GFP vector was constructed by linear plus linear homologous recombination (LLHR, Zhang *et al*., 1998). The linear pBBR1 vector was amplified with primers containing 25‐bp homology arms flanking the GFP/Pgenta promoter at their 5′ ends and 25‐bp PCR primers at their 3′ ends. Thus, the GFP/Pgenta promoter PCR products contain 25‐bp homology arms and 25‐bp PCR primers. Three PCR products were co‐electroporated by electroporator (1250 V, 5 ms, Eppendorf Corporation) into *E. coli* GB05‐dir (arabinose‐inducible, RecET‐expressing *E. coli* cells for LLHR) to construct the pBBR1‐kan‐Pgenta‐GFP. The recombinants were selected in LB medium with gentamicin at 4 μg ml^−1^. Subsequently, the construct was electroporated (1250 V, 5 ms, Eppendorf Corporation) into the Pf‐5, *retS* mutant, Pf‐5‐*nif* and *retS* mutant‐*nif* strains, separately.

### Quantitative analysis and isolation of antibiotics with HPLC

Cultures of the Pf‐5, *retS* mutant, Pf‐5‐*nif* and *retS* mutant‐*nif* strains were grown in KB medium with shaking at 150 r.p.m. for 24, 48 and 72 h, individually. The supernatants were collected and antibiotics extracted with ethyl acetate from the culture suspension were analysed using Thermo Scientific Dionex Ultimate 3000 system coupled to Bruker Amazon SL Ion Trap mass spectrometer (Bruker Corporation, Germany) with Thermo Scientific™ Acclaim™ C18 column (2.1 mm × 100 mm, 2.2 μm) at 30°C. The mobile phase contained of 0.1% aqueous acetic acid (solvent A) and acetonitrile (solvent B) with a linear gradient from 5% to 95% solvent B over 20 min, followed by 5 min at 95% solvent B. The flow rate was set to 0.5 ml min^−1^ and DAD light detector monitored at 250, 270, 290 and 310 nm respectively (**C1**, 254 nm; **C2**, 2,4‐DAPG, 270 nm; Xie *et al*., 2016). The experiment was repeated at least three times.

The ethyl acetate extracts of 6‐l culture were chromatographed using LC 3000 (Beijing Chuangxintongheng Science and Technology Co., Ltd) equipped with a C18‐reversed phase column (36 mm × 460 mm) using linear gradient elution with H_2_O‐MeOH (20:80) to 100% MeOH with 0.1% formic acid at a flow rate of 20 ml min^−1^, and then the fractions were detected with LC‐MS. Fractions containing **C2** were further chromatographed with the LC 3000 on C18 column (YMC‐Pack ODS‐A, 20 mm × 250 mm, 5 μm) at gradient from 65% to 76% (MeOH/H_2_O with 0.1% formic acid) for 50 min. Fractions containing **C1** was further chromatographed with Agilent 1260 Infinity using a C18 column (Agilent Co., USA; ZORBAX SB C18, 9.4 mm × 250 mm, 5 μm) with linear gradient elution from 22%–29% for 25 min.

### Bioassay for antifungal activity

The antifungal activity was assessed with *Rhizoctonia solani* as the indicator as described (Humair *et al*., 2009) on PDA medium plates. A mycelial plug (1 cm in diameter) from the actively growing fungus was placed in the centre of the plate. Cultures of Pf‐5, the *retS* mutant and Pf‐5 *nif* strains (20 μl; OD_600_ = 1.0) were, respectively, inoculated at four equidistant positions on the surface‐dry agar plates. After 5 days of incubation at 25°C, when the mycelium in control plates reached the edge of the plate, the inhibition zones were photographed.

The susceptibility of *Rhizoctonia solani* to **C1** and **C2** was analysed by mycelial plugs in 6‐well plates. Purified **C1** and **C2** were diluted in 7.5 ml PDA in each well (2–5) with concentrations of: 5, 10, 15, 50 and 100 μg ml^−1^. In the first well, ethanol was used as control. Plugs of 0.5 mm in diameter were then placed in the centre of each well and incubated at 25°C. When the mycelium in the control plates reached the edge of the plate, the zones of inhibition were photographed. This experiment was performed in triplicates.

### Transposition of the *nif* genes

A BAC vector containing the *nif* genes and a conjugation–transposition cassette was constructed via direct clone and Red/ET recombineering (Wang *et al*., 2016, 2017) and identified by *Kpn*I. The BAC vector was electroporated (1250 V, 5 ms, Eppendorf Corporation) into *E. coli* ET12567/pUZ8002, the donor strain for intergeneric conjugation with *P. protegens* pf‐5. The constructs were then introduced into the chromosome of *P. protegens* by conjugation, as described previously (Fu *et al*., 2008). *E. coli* containing the BAC vector was grown in LB broth at 37°C overnight with kanamycin at 50 μg ml^−1^ and gentamicin at 4 μg ml^−1^. *P. protegens* Pf‐5 was grown in LB broth overnight at 28°C with shaking. The *E. coli*–*P. protegens* conjugation mixture was plated on PMM agar supplemented with 20 mM MgSO_4_ and 15 μg ml^−1^ gentamicin and 100 μg ml ampicillin (to repress the growth of *E. coli* rather than the Pf‐5). Proper recombinant transformants were verified by PCR amplification using the check primer pairs, which were randomly designed along the *nif* genes with distance about 15–30 kb between primer pairs.

### Nitrogenase activity and ammonium concentration

For the nitrogenase activity assays, the acetylene reduction method was used as described previously (Brencic *et al*., 2009). After overnight incubation on LB plate, a single colony was grown in liquid medium for 8 h at 28°C. Cells were collected by centrifugation 5000 r.p.m. and 4°C for 10 min, washed three times with a 0.85% NaCl solution and then resuspended in nitrogen deficient medium to a final OD_600_ of 1.0. Two millilitre cultures of each culture were added to 18 ml of N‐free L media (Setten *et al*., 2013) in 100 ml flasks with a rubber stopper. Air in the flasks was replaced with argon containing 1% oxygen. After incubation for 6 h at 28°C with shaking at 250 r.p.m., 10% C_2_H_2_ was injected into the flasks. The cultures were then incubated for a further 4 h, and the gas phase was analysed using a Shimadzu GC2010 gas chromatograph to quantify ethylene production. The protein content was determined by the Bradford method (Bradford, 1976). For the ammonium concentration assays, dinitrogen was used instead of argon followed by incubation for 24 h. Ammonium in the medium was analysed by adding 2.5 ml of each supernatant to a mixture containing phenol (0.1 ml), sodium nitropusside (0.1 ml) and oxidizing agents (sodium nitroprusside, sodium hydroxide and sodium hydrochlorite; 0.25 ml), mixed well and incubated for 1 h; the absorbance was measured at 640 nm.

### Localization via root colonization assays

Seeds were sterilized with 10% NaClO for 15 min and then washed with ddH_2_O 5–6 times to remove the NaClO. Surface‐sterilized cucumber/wheat seeds were incubated with 1/2× MS solution for 24 h at 25°C before inoculation. Surface‐sterilized *Arabidopsis thaliana* seeds were vernalized for 3 days at 4°C in darkness before inoculation. For inoculation, seeds were treated with a bacterial suspension at 10^8^ CFU ml^−1^ (colony‐forming units) for 10 min and placed on filter papers for 2 min to remove any excess bacterial suspension. Then, the seeds were transferred to a flask (3 cm in diameter and 10 cm in height) filled with 15 ml of 1/2× MS semisolid agar medium with sucrose (30 g l^−1^). Plants were grown in a growth chamber with a photoperiod of 16 h light/8 h dark, at 80 μmol photons m^−2^ s^−1^ and a constant temperature of 25°C. Fluorescence was examined after 5–10 days of incubation with a Zeiss inverted fluorescence microscope (Zeiss LSM 800 Airyscan, Carl Zeiss Microimaging Inc., NY, USA) at 510–535 nm for GFP.

### Phenotype analysis of *Arabidopsis thaliana*

For the inoculation assays, *Arabidopsis thaliana* seeds were surface sterilized with 10% NaClO for 15 min and washed with ddH_2_O 5–6 times to remove the NaClO. Then, the seeds were vernalized for 3 days at 4°C in darkness. After rooted in the 1/2× MS semisolid agar medium with sucrose (30 g l^−1^), the seedlings were transferred to cultures containing a mixture of peat (a black substance rich in nutrient to help plants growth) and vermiculite (as a bedding medium to hold air and water for plants; 3:1 v/v), or, 100% vermiculite. After grown in 1/4 Hoagland's nutrient solution with or without nitrogen for 7 days, *Arabidopsis* seedlings were inoculated with overnight cultures of bacteria with or without the *nif* genes that were grown at 28°C, washed with saline solution twice and then resuspended to OD_600_ = 0.3 (≈10^8^ CFU ml^−1^). One millilitre of the bacterial solution was added on the soil surface at a distance of approximately 0.5 cm away from the roots. *Arabidopsis* seedlings were then grown in a growth room at 22°C and 120 μmol photons m^−2^ s^−1^ with a photoperiod of 16 h light/8 h darkness.

### Phenotype analysis of the engineered *P. protegens* Pf‐5 on wheat and cucumber

*Rhizoctonia solani* was grown on PAD plates for 7 days at 25°C. Ten mycelial plugs (1‐cm‐diameter) from the actively growing fungus were placed into 10 g ground organically grown processed oats and incubated for 14 days at 25°C. After incubation, the 10 g oats incubated *Rhizoctonia solani* containing oospores and hyphae were mixed with 90 g soil (peat: vermiculite, 3:1 v/v). Prior to the transplanting, inoculation of plants with *Rhizoctonia solani* was performed by placing the homogenized *Rhizoctonia solani* soil onto the surfaces at the height of 1 cm.

Surface‐sterilized cucumber/wheat seeds were incubated in 1/2× MS semisolid agar medium with sucrose (30 g l^−1^). Under pathogen and N‐limited double treatment condition, 2 weeks old rooted wheat/cucumber seedlings were transferred to each pot for 3 days of cocultivation. Cultures of Pf‐5, the *retS* mutant, Pf‐5 *nif* and the *retS* mutant‐*nif* were, respectively, inoculated as described above. The plants were irrigated with 1/4 Hoagland's nutrient solution without nitrogen and were grown in a growth room at 25°C and 120 μmol photons m^−2^ s^−1^, 16 h light/8 h dark. Photographs were taken after 14 days after inoculation treatment, and the fresh weights were measured.

### Statistical analysis

All experiments data were analysed by GraphPad Prism 6 (GraphPad Software, Inc., San Diego, CA, USA). *P < 0.05, **P < 0.01, ***P < 0.001 and ****P < 0.0001 according to ANOVA with Dunnett as post doc test.

## Acknowledgements

The plant fungal were supported by the Qingdao Zhongda Agricultural Science and Technology Co., Ltd. Thanks for the generous gift. In addition, we acknowledge financial support by the Program of Global Experts (1000 plan), the Project of Taishan industry leading talent in Shandong province (LJNY201603), the Program of Introducing Talents of Discipline to Universities (B16030), the Major Basic Program of Natural Science Foundation of Shandong Province (ZR2017ZB0212), Natural Science Foundation of Shandong Province (ZR201709210045), National Natural Science Foundation of China (31700004) and Natural Science Foundation of Jiangsu Province (BK20160368).

## Conflicts of interest

The authors declare that they have no competing interests.

## Author contributions

Xiaoshu Jing designed and performed the experiments, analysed the experimental data and prepared the manuscript. Qingwen Cui, Xiaochen Li, Jia Yin and Deng Pan partly participated in the experiments. *Vinothkannan Ravichandran* proofread this manuscript. Jun Fu, Qiang Tu, Hailong Wang, Xiaoying Bian and Youming Zhang conceived research plan. All authors have read and approved the final version of this manuscript.

## Supporting information


**Table S1.** Secondary metabolite clusters prediction in *P. protegens* Pf‐5 and DSM4166 based on GenBank sequences and antiSMASH analysis.**Table S2.** All the strains, plasmid and primers used in this paper.**Fig. S1.** HPLC spectrum of secondary metabolites of Pf‐5 and the *retS* mutant from cultures of 24 h (A) 48 h (B) and 72 h (C).**Fig. S2.** Isolation of C1 and C2 by preparative HPLC.**Fig. S3.** MS spectrum, UV visible spectrum and chemical structure of pyoluteorin.**Fig. S5.** Graphical explanation of the *retS* mutant‐*nif* engineering.**Fig. S4.** MS spectrum, UV visible spectrum and chemical structure of orfamide.**Fig. S6.** Growth rates of Pf‐5, *retS* mutant, Pf‐5‐*nif* and *retS* mutant‐*nif*.**Fig. S7.** Ammonium production assays in *P. protegens*.**Fig. S8.** Control fluorescence micrographs images using a confocal laser scanning microscope. (A) Construct and fluorescence micrographs of GFP‐expressing vector in *P. protegens* under the control of constitutive promoters. (B) Fluorescence micrographs of the wheat, cucumber and *Arabidopsis* roots inoculated with the unmodified strain.**Fig. S9.** Growth phenotype of *Arabidopsis* on 100% vermiculite. *Arabidopsis* was grown in cultures containing 100% vermiculite without nitrogen.**Fig. S10.** Growth phenotype of cucumber (A–D) and wheat (E–H) under N limited condition. A–H show the plant performance of cucumber (A) and wheat (E), and the fresh weights of seedlings (B, F), shoots (C, G) or roots (D, H). Each column is the mean of 6 independent measurements. Bars represent standard error of the mean. Bars scale 5 cm (A), 2 cm (D).
