# Supplementary figures and images for: Engineering Pseudomonas protegens Pf‐5 to improve its antifungal activity and nitrogen fixation
Source: Microb Biotechnol. 2018 Nov 20;13(1):118–33. doi: 10.1111/1751-7915.13335 (PMC6984399; doi:10.1111/1751-7915.13335)

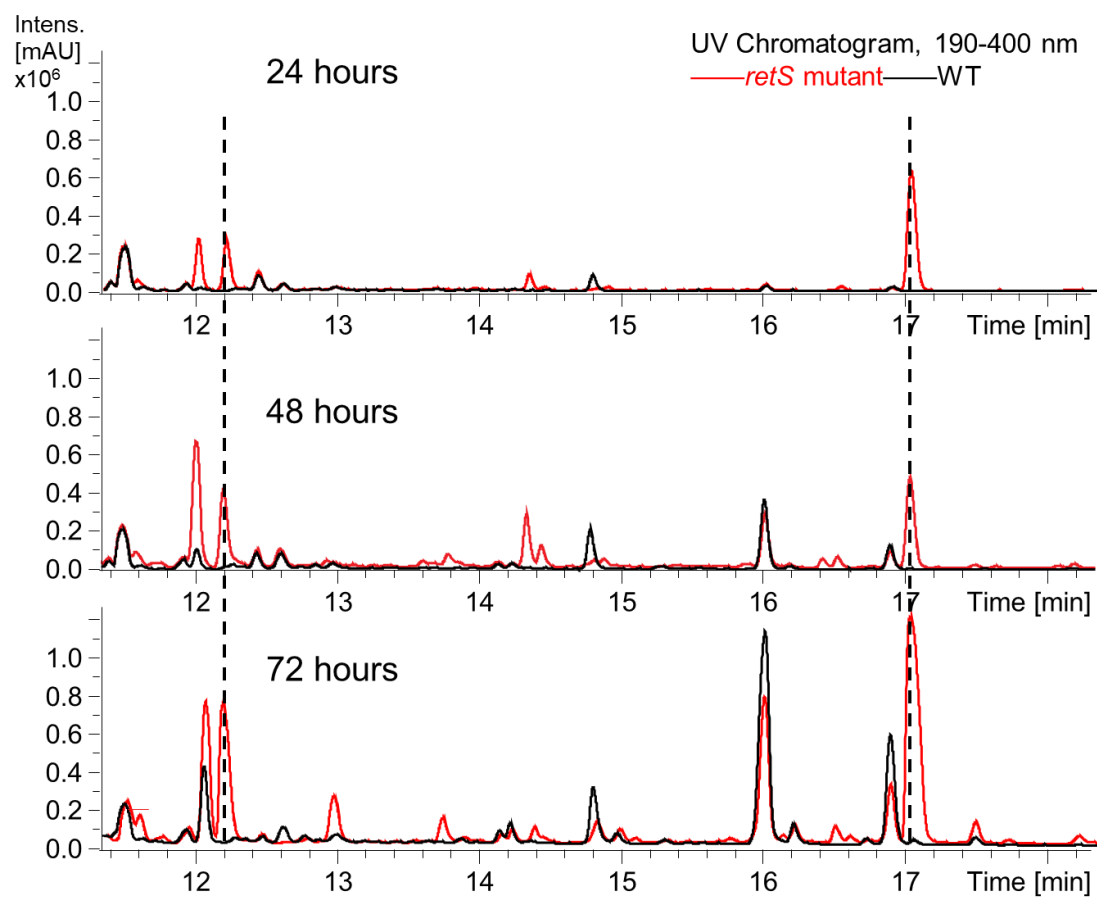

Figure S1

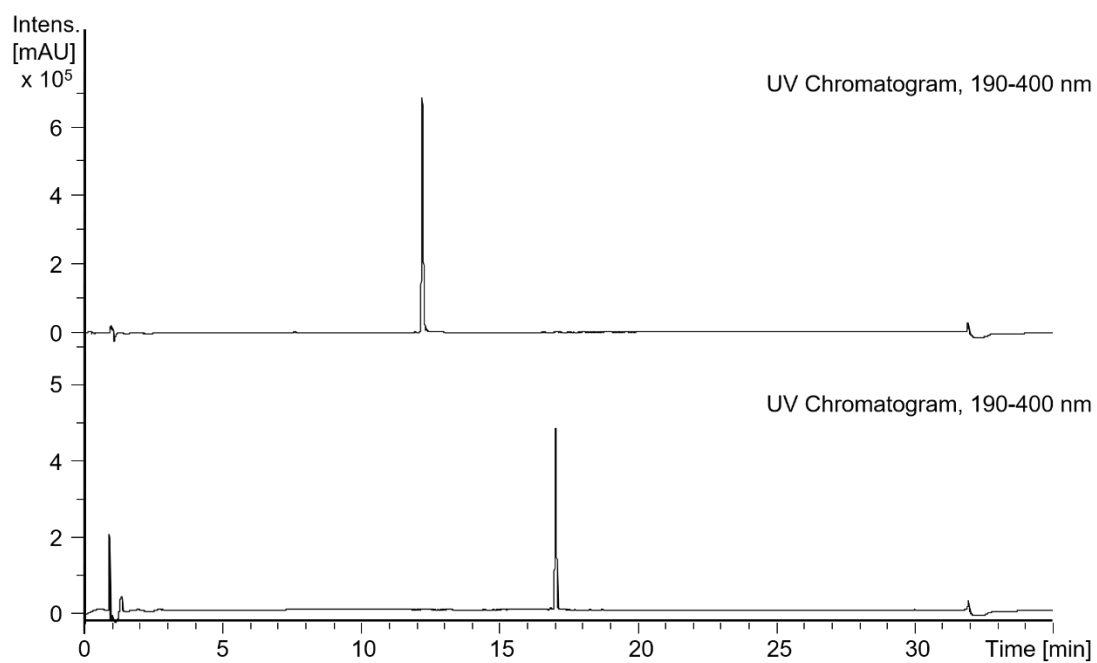

Figure S2

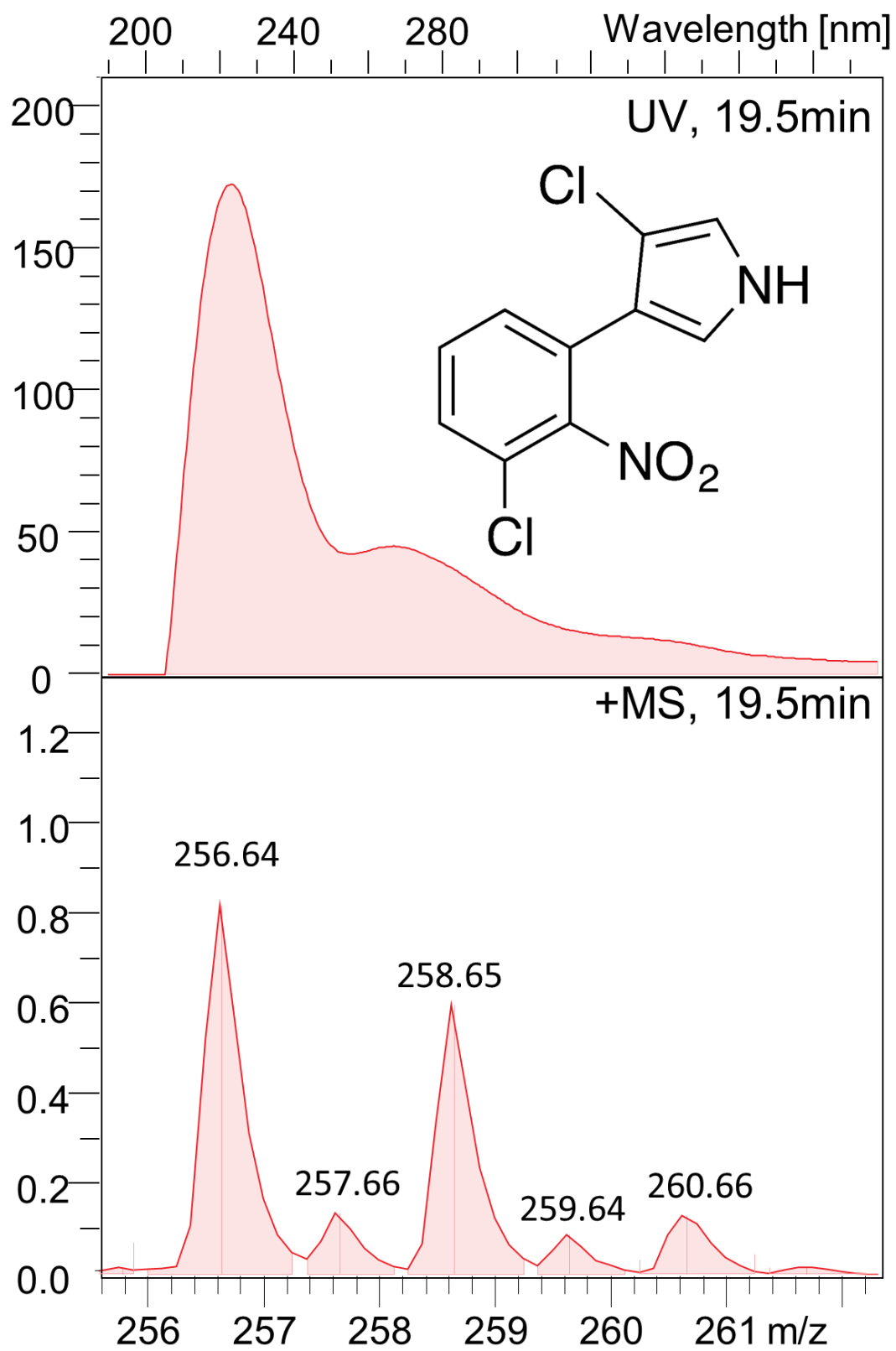

Figure S3



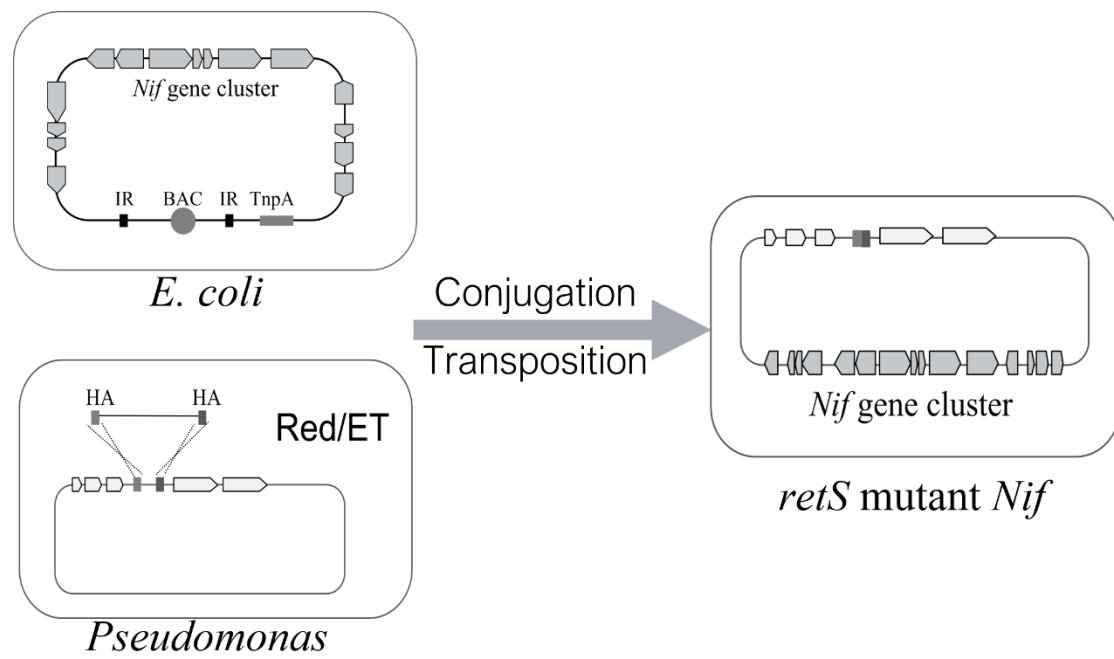

Figure S5

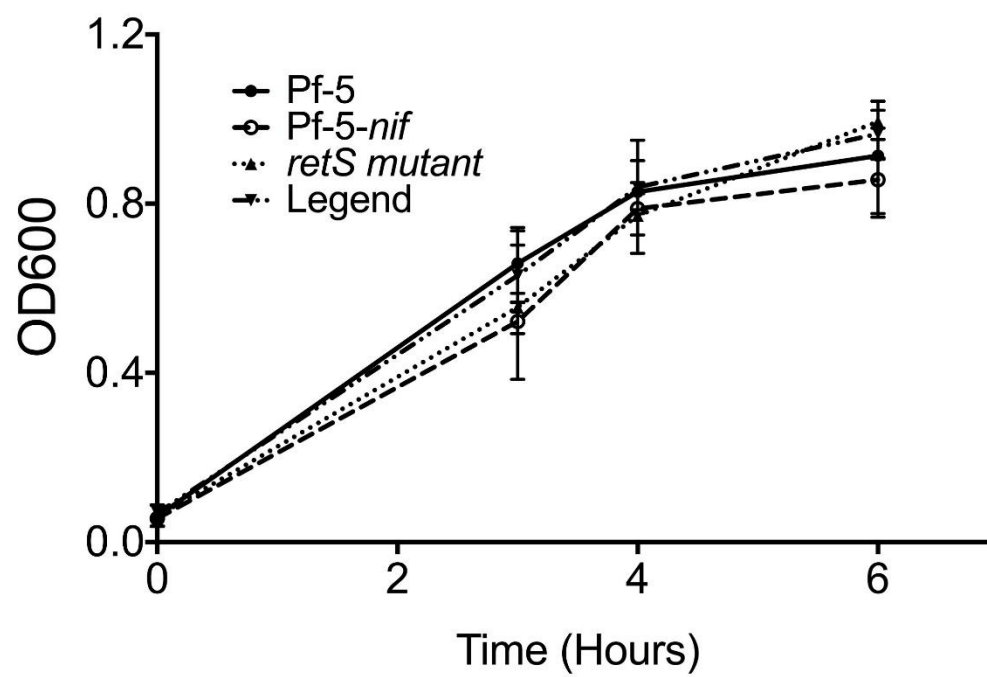

Figure S6

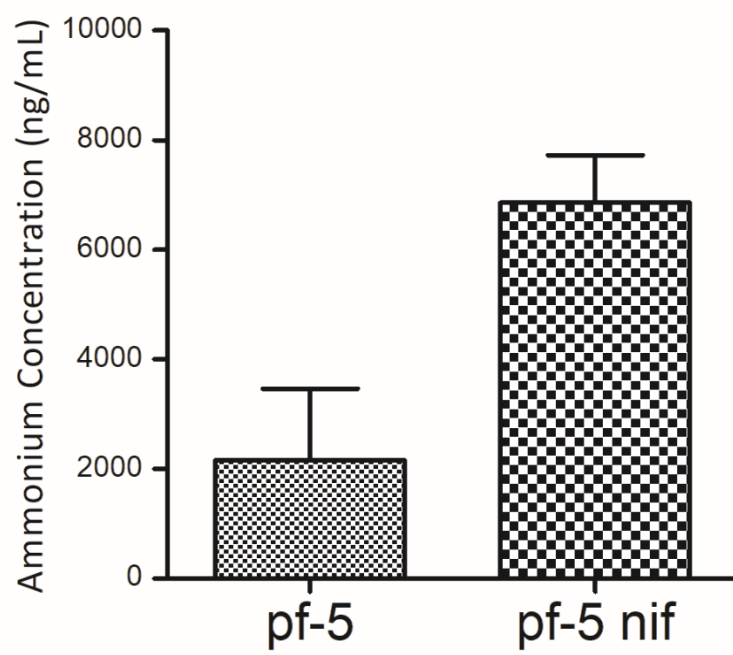

Figure S7

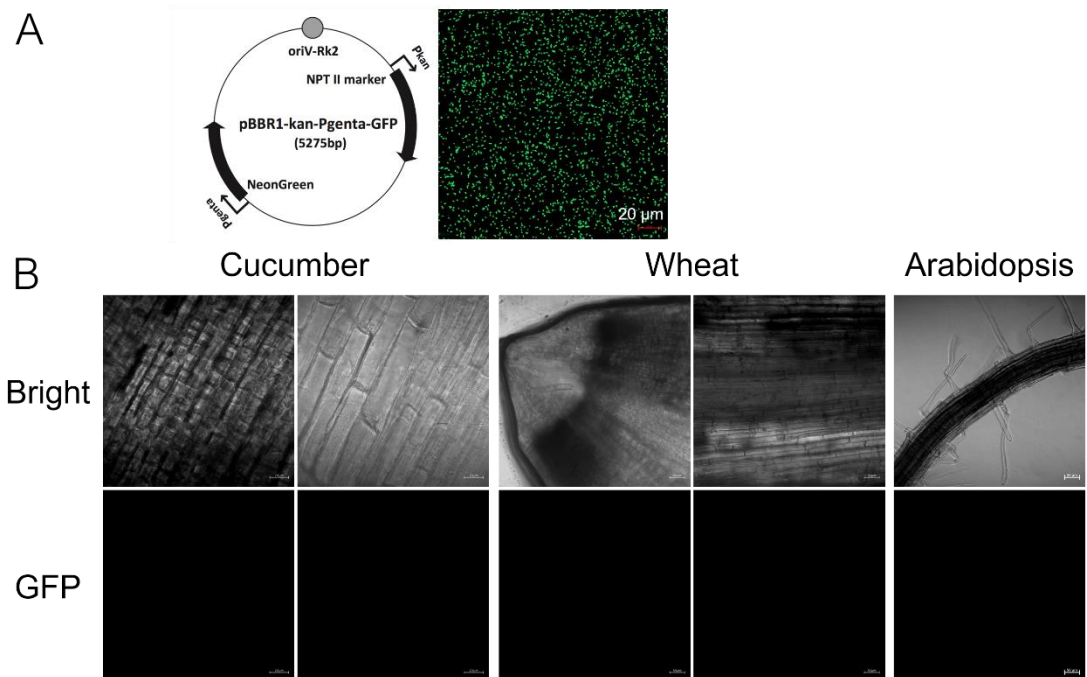

Figure S8

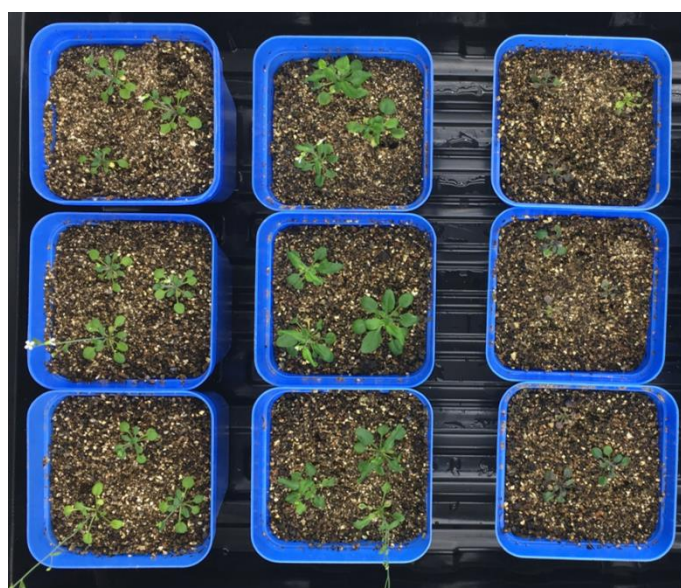

+N

Nif

*retS* mutant

Figure S9

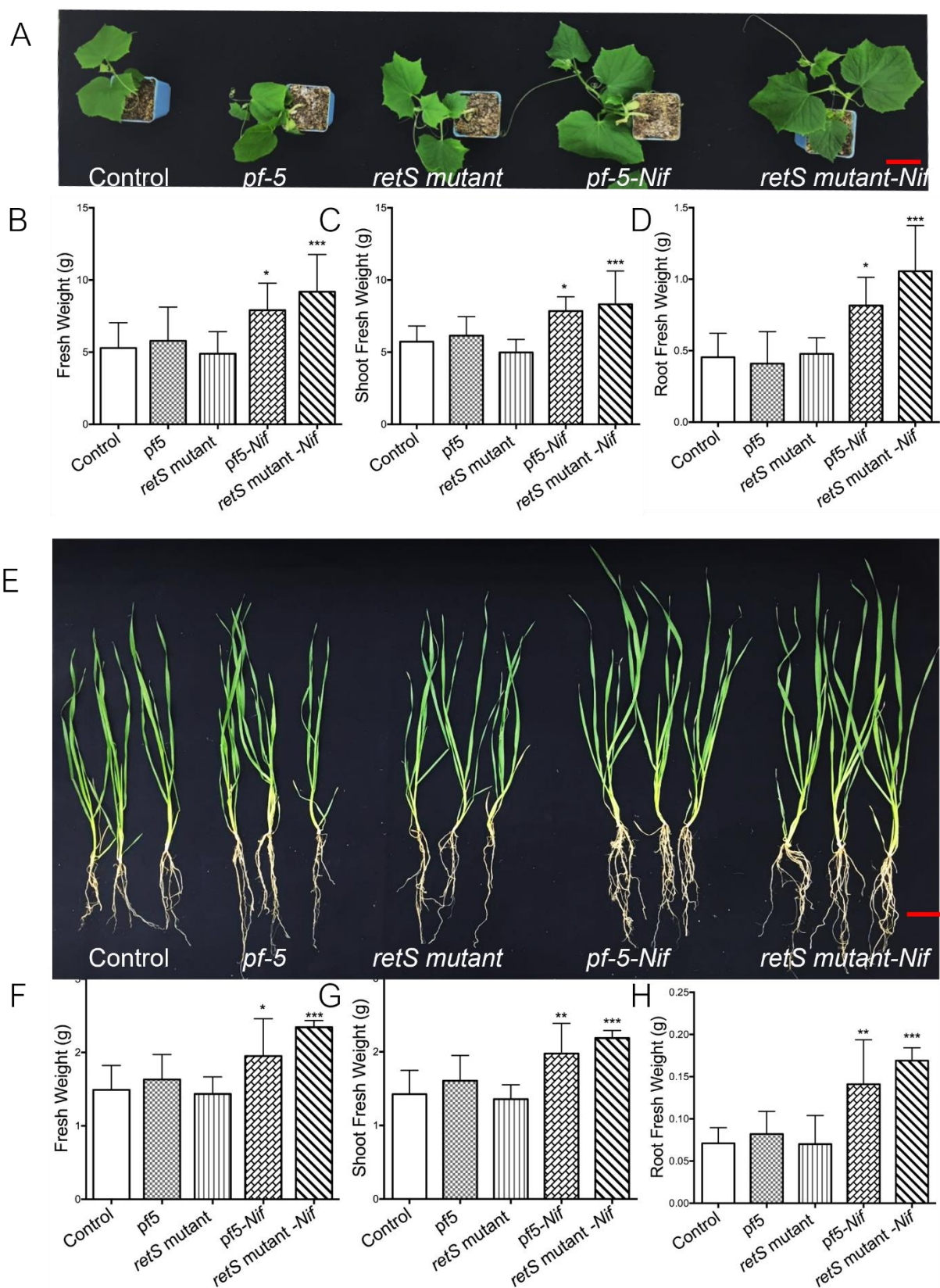

Figure S10

Supplement: Supplementary file 1 — Table S1. Secondary metabolite clusters prediction in P. protegens Pf‐5 and DSM4166 based on GenBank sequences and antiSMASH analysis.Table S2. All the strains, plasmid and primers used in this paper.Fig. S1. HPLC spectrum of secondary metabolites of Pf‐5 and the retS mutant from cultures of 24 h (A) 48 h (B) and 72 h (C).Fig. S2. Isolation of C1 and C2 by preparative HPLC.Fig. S3. MS spectrum, UV visible spectrum and chemical structure of pyoluteorin.Fig. S5. Graphical explanation of the retS mutant‐nif engineering.Fig. S4. MS spectrum, UV visible spectrum and chemical structure of orfamide.Fig. S6. Growth rates of Pf‐5, retS mutant, Pf‐5‐nif and retS mutant‐nif.Fig. S7. Ammonium production assays in P. protegens.Fig. S8. Control fluorescence micrographs images using a confocal laser scanning microscope. (A) Construct and fluorescence micrographs of GFP‐expressing vector in P. protegens under the control of constitutive promoters. (B) Fluorescence micrographs of the wheat, cucumber and Arabidopsis roots inoculated with the unmodified strain.Fig. S9. Growth phenotype of Arabidopsis on 100% vermiculite. Arabidopsis was grown in cultures containing 100% vermiculite without nitrogen.Fig. S10. Growth phenotype of cucumber (A–D) and wheat (E–H) under N limited condition. A–H show the plant performance of cucumber (A) and wheat (E), and the fresh weights of seedlings (B, F), shoots (C, G) or roots (D, H). Each column is the mean of 6 independent measurements. Bars represent standard error of the mean. Bars scale 5 cm (A), 2 cm (D). [file MBT2-13-118-s001.pdf]
